# Investigating the Photodissociation Dynamics of CF_2_BrCF_2_I in CCl_4_ through Femtosecond Time-Resolved Infrared Spectroscopy

**DOI:** 10.3390/ijms24021319

**Published:** 2023-01-10

**Authors:** Seongchul Park, Juhyang Shin, Manho Lim

**Affiliations:** Department of Chemistry and Chemistry Institute for Functional Materials, Pusan National University, Busan 46241, Republic of Korea

**Keywords:** femtosecond vibrational spectroscopy, conformation-dependent dynamics, secondary reaction, CF_2_BrCF_2_Br production, reaction dynamics in solution

## Abstract

The photodissociation dynamics of CF_2_BrCF_2_I in CCl_4_ at 280 ± 2 K were investigated by probing the C−F stretching mode from 300 fs to 10 μs after excitation at 267 nm using time-resolved infrared spectroscopy. The excitation led to the dissociation of I or Br atoms within 300 fs, producing the CF_2_BrCF_2_ or CF_2_ICF_2_ radicals, respectively. All nascent CF_2_ICF_2_ underwent further dissociation of I, producing CF_2_CF_2_ with a time constant of 56 ± 5 ns. All nascent *g*-CF_2_BrCF_2_ isomerized into the more stable *a*-CF_2_BrCF_2_ with a time constant of 47 ± 5 ps. Furthermore, *a*-CF_2_BrCF_2_ underwent a bimolecular reaction with either itself (producing CF_2_BrCF_2_Br and CF_2_CF_2_) or Br in the CCl_4_ solution (producing CF_2_BrCF_2_Br) at a diffusion-limited rate. The secondary dissociation of Br from *a*-CF_2_BrCF_2_ was significantly slow to compete with the bimolecular reactions. Overall, approximately half of the excited CF_2_BrCF_2_I at 267 nm produced CF_2_BrCF_2_Br, whereas the other half produced CF_2_CF_2_. The excess energies in the nascent radicals were thermalized much faster than the secondary dissociation of I from CF_2_ICF_2_ and the observed bimolecular reactions, implying that the secondary reactions proceeded under thermal conditions. This study further demonstrates that structure-sensitive time-resolved infrared spectroscopy can be used to study various reaction dynamics in solution in real time.

## 1. Introduction

Vicinal dihaloethanes are dissociated using ultraviolet (UV) energy to form haloethyl radicals that undergo various secondary reactions [1,2,3,4,5,6,7,8,9,10,11,12]. Significant efforts have been made to understand the dynamics of the photodissociation of vicinal dihaloethanes, including the reaction dynamics of haloethyl radicals, in solution and in the gas phase [1,2,3,4,5,6,7,8,9,10,11,12]. The initial reaction of vicinal dihaloalkanes, in which one halogen atom is immediately dissociated upon UV excitation to form haloethyl radicals, shows similar characteristics in the gas and condensed phases [5,8,10]. However, the secondary reactions of haloethyl radicals in solution show different kinetics from the gas-phase reaction depending on the type of substituted halogen atom and the spatial isomers of haloethyl radicals [8,10].

Vicinal diiodoethanes, such as CH_2_ICH_2_I, CF_2_ICH_2_I, and CF_2_ICF_2_I, were mainly investigated because the iodoethyl radicals produced upon UV excitation contain weak C−I bonds that readily proceed with secondary dissociation [1,2,3,5,7,8,10,12]. In the case of the photodissociation of CF_2_ICF_2_I in the gas phase, a fraction of the iodoethyl radicals dissociate the secondary C−I bond with a time constant of 26 ± 7 ps to form CF_2_CF_2_ and I [2]. Secondary dissociation of haloethyl radicals often occurs because of the weakness of the halogen–carbon bonds; however, these bonds strengthen upon fluorination. The C−I bond is weaker in CH_2_ICH_2_ than in CF_2_ICF_2_ because the π-bond formed concertedly upon the fission of the C−I bond is stronger in the case of CH_2_ICH_2_ [7]. The primary C−I bond breaking of dihaloalkanes has two dissociation channels, leading to I(^2^P_1/2_) and I(^2^P_3/2_) [3,4,6,7,9]. The internal energy of haloethyl radicals is larger with the reaction channel of I(^2^P_3/2_); thus, the haloethyl radicals formed with I(^2^P_3/2_) undergo faster and more efficient secondary dissociation in the gas phase [3,4,6,7,9]. 

In the condensed phase, CF_2_ICF_2_ radicals are also formed by the UV excitation of CF_2_ICF_2_I [5,8,10]. However, the secondary reaction of CF_2_ICF_2_ radicals in solution exhibits very different kinetics from those of gas-phase reactions, depending on their stereochemical characteristics and the surrounding solvents. CF_2_ICF_2_I in solution has two stereoisomers, *anti* and *gauche*, and the corresponding CF_2_ICF_2_ radicals formed from these isomers are *a*-CF_2_ICF_2_ and *g*-CF_2_ICF_2_, respectively [13]. According to the time-resolved infrared (TRIR) spectroscopy experiments [8], all the nascent *g*-CF_2_ICF_2_ produced upon 267 or 310 nm excitation reacted with the dissociated I atom within 1 ps to form I_2_∙∙C_2_F_4_—a weakly associated iodine tetrafluoroethylene complex. Furthermore, some of the nascent *a*-CF_2_ICF_2_ also reacted with an I atom with a time constant of 1.5 ps to form I_2_∙∙C_2_F_4_. The formed I_2_∙∙C_2_F_4_ dissociated with a time constant of 180 ± 5 ps in CCl_4_ and 46 ± 3 ps in CH_3_CN and CD_3_OH to produce CF_2_CF_2_ + I_2_. The remaining *a*-CF_2_ICF_2_ in solution underwent secondary dissociation with a time constant of 3–6 ns to produce CF_2_CF_2_. As all of the excess internal energy in the haloethyl radicals dissipates into the solvent on the picosecond time scale [8], the haloethyl radicals in solution are in thermal equilibrium when they dissociate the second I atom, unlike the reaction in the gas phase. Consequently, the kinetics related to the two dissociation channels, I(^2^P_1/2_) and I(^2^P_3/2_), were not exhibited in the photodissociation of CF_2_ICF_2_I in solution [8]. 

The CF_2_ICF_2_ radical in solution has rich reaction dynamics; however, it exhibits only a simple dissociative reaction of the weak C−I bond on the nanosecond time scale. The C–I bond has a lifetime of a few nanoseconds; thus, any reaction longer than that, such as a bimolecular reaction (BR) involving the radical, cannot be observed unless the concentration of the radical is sufficiently high to expedite the BR. Haloethyl radicals with more stable C−X bonds, such as C−Br or C−Cl, can survive sufficiently long to participate in a BR involving the radical and to exhibit reactions other than the simple dissociation of the C−X bond.

By dissociating I or Br atoms, UV-excited CF_2_BrCF_2_I can generate CF_2_BrCF_2_ or CF_2_ICF_2_ radicals, respectively [1,2,3,7,9,12]. In the gas phase, the photodissociation of CF_2_BrCF_2_I at 248 or 266 nm produces CF_2_BrCF_2_, while photodissociation at 193 nm produces 63% CF_2_BrCF_2_ and 37% CF_2_ICF_2_ [4]. The Br-dissociated radical CF_2_CH_2_I was produced by the 266 nm excitation of CF_2_BrCH_2_I in the gas phase [6], suggesting that the primary C−Br dissociation is feasible through 266 nm excitation. The secondary reaction of CF_2_ICF_2_ is well established in solution, as well as in the gas phase; however, the available spectroscopic and dynamical information on CF_2_BrCF_2_ is scarce [7,14]. Akesson and coworkers investigated the photodissociation dynamics of CF_2_BrCF_2_I in solution after excitation at 266 nm by probing the formation of IBr using time-resolved electronic spectroscopy [10]. The haloethyl radical was not monitored, but its formation and dynamics were conjectured by the kinetics of the electronic absorption signal of IBr. This is the only reported time-resolved study on CF_2_BrCF_2_I.

Recently, we measured the photodissociation dynamics of CF_2_BrCF_2_I in CCl_4_ at 280 K from 0.3 to 320 ps after excitation with a 267 nm pulse to study the rotational isomerization of the haloethyl radical about the C−C bond [15]. The 267 nm excitation immediately dissociates Br or I atoms from CF_2_ICF_2_Br in CCl_4_ at 280 K, producing nascent radicals consisting of 33 ± 3% *a*-CF_2_BrCF_2_, 49 ± 3% *g*-CF_2_BrCF_2_, and 18 ± 3% *a*-CF_2_ICF_2_ [15]. While no geminate rebinding of the dissociated halogen atom with nascent radicals or secondary reactions of *a*-CF_2_ICF_2_ occur within 320 ps, almost all *g*-CF_2_BrCF_2_ rotationally isomerizes into the more stable *a*-CF_2_BrCF_2_ with a time constant of 47 ± 5 ps [15]. The excess energy in the nascent radical is thermalized with a time constant of 15 ± 3 ps [15], implying that any secondary reaction proceeding slower than 15 ps becomes a reaction in thermal equilibrium. The radicals *a*-CF_2_BrCF_2_ and *a*-CF_2_ICF_2_ survive long after rotational isomerization, and the former is expected to survive sufficiently long to participate in a BR involving the radical. Therefore, comparative reaction dynamics between the two radicals can be probed by observing the entire reaction dynamics of CF_2_BrCF_2_I excited at 267 nm in a CCl_4_ solution. Owing to the sensitivity of IR spectroscopy to molecular structure, the conformer-specific reactions of the radicals obtained from the photodissociation of CF_2_BrCF_2_I in solution can be observed in detail, similar to the case of the photodissociation of CF_2_ICF_2_I in solution [8]. 

In this study, we investigated all the subsequent reactions after the photodissociation of CF_2_BrCF_2_I in a CCl_4_ solution at 267 nm through TRIR spectroscopy for up to 10 μs. With the support of quantum-chemical calculations and available spectra, all the transient absorptions in the TRIR spectra of CF_2_BrCF_2_I in CCl_4_ were assigned, and their kinetics were determined. Upon excitation, CF_2_BrCF_2_I immediately produces *a*-CF_2_BrCF_2_, *g*-CF_2_BrCF_2_, and *a*-CF_2_ICF_2_ [15]. Although *a*-CF_2_ICF_2_ undergoes a simple dissociation of the C−I bond, most of the *g*-CF_2_BrCF_2_ isomerize into *a*-CF_2_BrCF_2_ which undergoes a bimolecular reaction with itself or the dissociated Br atom in the solution. Structure-sensitive TRIR spectroscopy enabled us to determine the real-time reaction dynamics of UV-excited CF_2_BrCF_2_I in CCl_4_ at 280 K.

## 2. Results and Discussion

**Figure 1:**
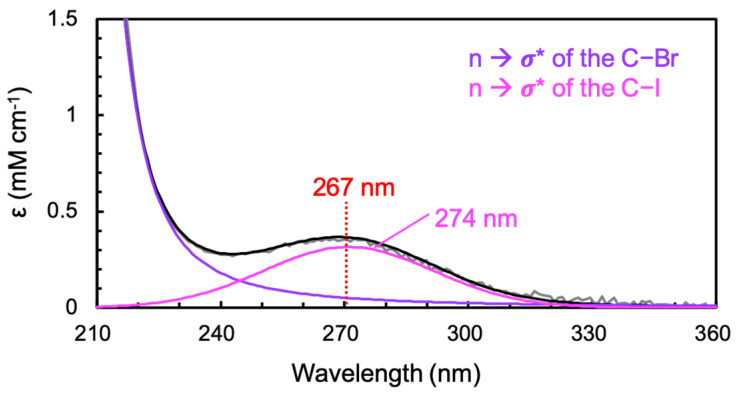
Equilibrium UV–Vis spectrum of CF_2_BrCF_2_I dissolved in CCl_4_ at room temperature. The electronic spectrum (gray line) is decomposed into the absorption bands for the n → σ* transitions of the C−Br bond (purple line) and the C−I bond (magenta line) at 274 nm. The dotted red line positioned at 267 nm was the excitation wavelength in our experiments.

The equilibrium electronic spectrum of CF_2_BrCF_2_I dissolved in CCl_4_ showed a weak absorption band at 274 nm and a strong band above 200 nm (Figure 1), which was slightly red-shifted from the peaks at 268 and 193 nm in the gas phase [9]. The weak absorption at 274 nm, assigned to the n → σ* transition of the C−I bond, leads to the immediate dissociation of the I atom [1,2,3,12], while the strong absorption above 200 nm, assigned to the n → σ* transition of the C−Br bond, leads to the immediate dissociation of the Br atom [9]. As the wing of this strong absorption band extends up to 300 nm and accounts for 18 ± 3% of the absorption at 267 nm, the photoexcitation of CF_2_BrCF_2_I at 267 nm can lead to the dissociation of not only the I atom but also the Br atom. Specifically, 82 ± 3% of the absorption intensity at 267 nm arises from the weak absorption band at 274 nm; thus, excitation at 267 nm would promote 82 ± 3% (18 ± 3%) of CF_2_BrCF_2_I to the antibonding state of the C−I (C−Br) bond. Consequently, 82 ± 3% (18 ± 3%) of the excited CF_2_BrCF_2_I at 267 nm is expected to dissociate into CF_2_BrCF_2_ + I (CF_2_ICF_2_ + Br). The contribution from the strong band to the absorption at 267 nm in the gas phase was estimated to be 9 ± 4% [9], which would result in 9 ± 4% CF_2_ICF_2_ production upon excitation at 267 nm in the gas phase, which is smaller than that in CCl_4_. 

The TRIR spectra of CF_2_BrCF_2_I in CCl_4_ at 280 ± 2 K were measured in the spectral region of 1370−1020 (1020−870) cm^−1^ that covers all of the C−F stretching modes of the molecule over a broad time span from 0.3 ps to 10 μs (1 ns) after excitation at 267 nm, encompassing the entire excitation-induced reaction. As the photodissociation reaction was found to be complete at 1 µs in previous experiments on similar dihaloalkanes in solution [8,10], the time range of 0.3 ps to 10 μs was expected to be sufficient to observe the fates of all of the intermediates and products induced by the photoexcitation of CF_2_BrCF_2_I in CCl_4_. The absorption bands in the 1300−1100 cm^−1^ region were congested, while those in the 1020−870 cm^−1^ region (vide infra) were well resolved. Thus, the TRIR spectra in the 1020−870 cm^−1^ region were collected up to 1 ns to confirm the band assignment [15] and dynamics of the CF_2_BrCF_2_ radical (vide infra), even though this was time-consuming because of the inferior sensitivity of our TRIR spectrometer in such a long-wavelength spectral region.

**Figure 2:**
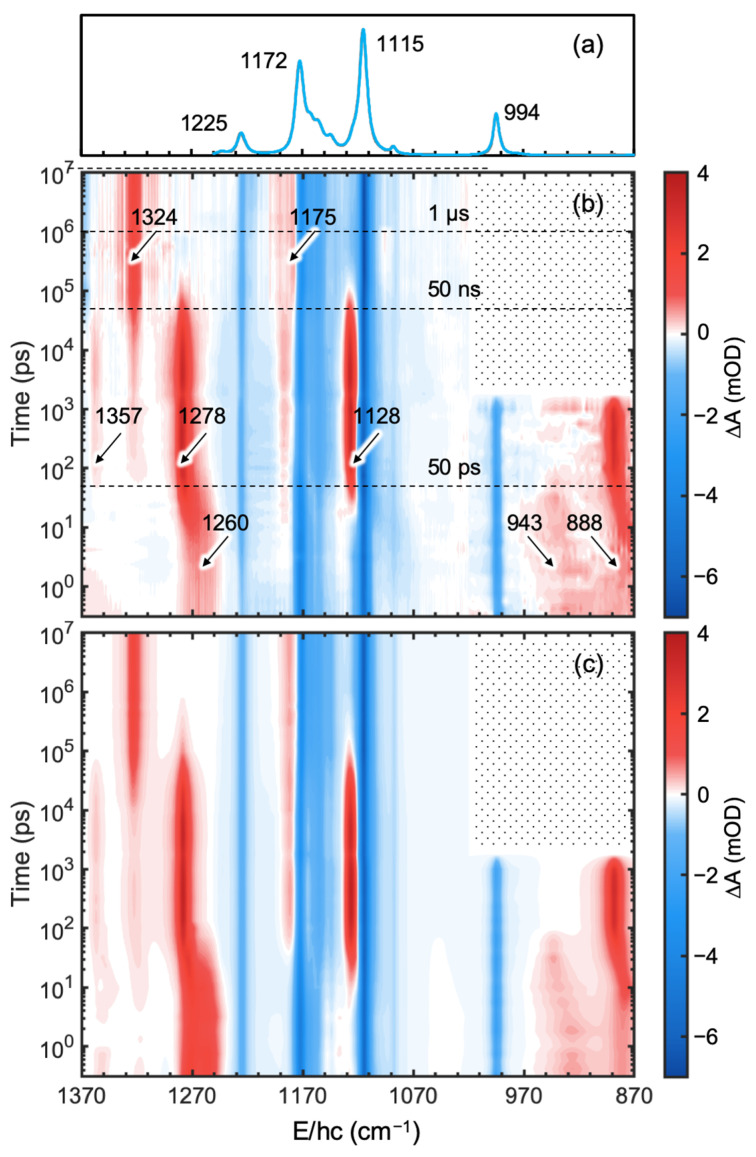
(**a**) Equilibrium FTIR spectra of CF_2_BrCF_2_I in CCl_4_ with the positions of four main bands. (**b**) Contour plot of the TRIR spectra of CF_2_BrCF_2_I in CCl_4_ at 280 ± 2 K obtained after excitation of CF_2_BrCF_2_I at 267 nm. The data in the spectral regions of 1370−1020 cm^−1^ and 1020−870 cm^−1^ were collected from 0.3 ps to 10 μs and from 0.3 ps to 1 ns, respectively. Apparent bands are marked by arrows, and their peak positions are also mentioned. Dotted lines at 50 ps, 50 ns, and 1 μs guide the eye to analyze the apparent kinetics of transient signals. The absorbance difference, ΔA, was obtained by subtracting the absorbance of the sample before excitation from that after excitation. The absorbance is given in optical density (OD), where 1 mOD = 10^−3^ OD. (**c**) Contour plot of the fitted TRIR spectra of CF_2_BrCF_2_I in CCl_4_ (see text).

As shown in Figure 2b, negative absorption bands (bleach) appeared immediately after excitation at the positions of the absorption bands of the equilibrium spectrum of CF_2_BrCF_2_I. The bleach signal arises from the depletion of the population of CF_2_BrCF_2_I in the ground state upon excitation. The four main absorption bands (negative absorption) of CF_2_BrCF_2_I near 1225, 1172, 1115, and 994 cm^−1^ appeared at 0.3 ps and maintained their magnitude up to 10 μs. This suggests that the photoreaction proceeds within 0.3 ps and none of the nascent photofragments return to the reactant for up to 10 μs. New absorption bands can be categorized into three groups according to their kinetics: (1) bands appearing immediately after excitation, (2) bands growing around 50 ps and decaying near 50 ns, and (3) bands growing at ~50 ns and maintaining their amplitudes up to 10 μs, which is the last pump-probe delay time in our experiment. This suggests that the nascent products may undergo secondary reactions to produce intermediates that can further react to generate the final photoproducts. The absorption bands immediately appearing after excitation (group (1)) were assigned to the nascent photoproducts [15] and those maintained up to 10 μs after growing near 50 ns to the final products produced by secondary reactions of the reaction intermediates. Absorption bands near 1278 and 1128 cm^−1^ grew near 50 ps and decayed around 50 ns; thus, they were assigned to the reaction intermediates that produced the final products through the secondary reactions. The immediately appearing absorption bands near 1260, 943, and 888 cm^−1^ were initially broad and shifted toward blue with time, suggesting that the nascent photofragments were produced with excess energy that relaxed via the anharmonically-coupled lower-frequency modes (thermal relaxation) [15,16,17,18]. The absorption bands at 1324 and 1175 cm^−1^, assigned to CF_2_CF_2_ in CCl_4_ [8,13,14,19], grew near 50 ns and maintained their intensity up to 10 μs, indicating that CF_2_CF_2_ is the final product formed in tens of nanoseconds.

**Figure 3:**
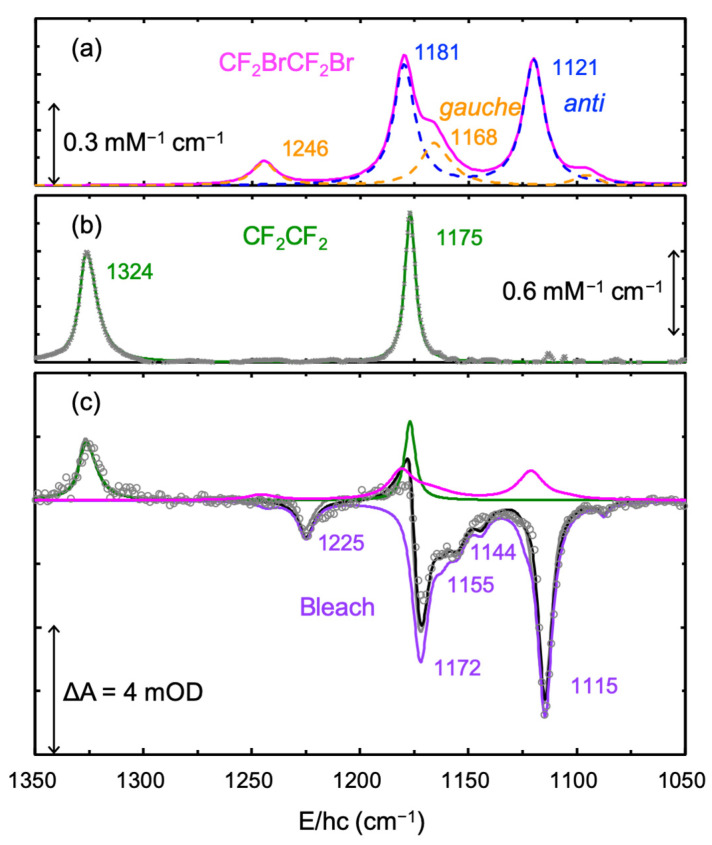
(**a**) Equilibrium FTIR spectrum of CF_2_BrCF_2_Br in CS_2_ which was obtained by fitting data in the NIST data base [20] (magenta line). The spectrum is well described as the sum of the absorption spectra of *a*-CF_2_BrCF_2_Br (dashed blue line) and *g*-CF_2_BrCF_2_Br (dashed orange line) using density functional theory (DFT) calculations. (**b**) Equilibrium FTIR spectrum of CF_2_CF_2_ in CCl_4_ [8] (crosses). The spectrum is well described by two absorption bands (green line). (**c**) Averaged spectrum (open circles) of the TRIR spectra of CF_2_BrCF_2_I from 1 to 10 μs overlapped with the equilibrium difference spectrum (gray line) obtained by subtracting the absorption spectrum measured before the pump-probe experiment from that after the experiment. The averaged spectrum corresponds well with the sum (black line) of the three equilibrium spectra of CF_2_BrCF_2_I (purple line), CF_2_CF_2_ (green line), and CF_2_BrCF_2_Br (magenta line). Positions of the bands are given in the figure.

Notably, TRIR spectra beyond 1 μs did not evolve with time, indicating the completion of the photoreaction. Thus, new absorption bands in the TRIR spectra beyond 1 μs should arise from the final products and bleach the signal from the depleted reactant. The TRIR spectra from 1 to 10 μs were averaged to obtain more reliable spectra for the final products. As shown in Figure 3c, the averaged TRIR spectrum overlapped well with the difference spectrum obtained by subtracting the absorption spectrum measured before the pump-probe experiment from that measured after the experiment, confirming that the photoreaction was complete by ~1 μs. Apart from the absorption spectrum of CF_2_CF_2_ and the inverted absorption spectrum of CF_2_BrCF_2_, the averaged TRIR spectrum contained an additional absorption spectrum, which was assigned to CF_2_BrCF_2_Br based on the reported spectrum of CF_2_BrCF_2_Br in CS_2_ (Figure 3a) [20]. Clearly, the photodecomposition of CF_2_BrCF_2_I leads to two products: CF_2_CF_2_ and CF_2_BrCF_2_Br. The decomposition of the averaged TRIR spectrum suggests that the photoexcitation of CF_2_BrCF_2_I in CCl_4_ at 267 nm produces CF_2_CF_2_ (50 ± 3%) and CF_2_BrCF_2_Br (50 ± 3%).

**Figure 4:**
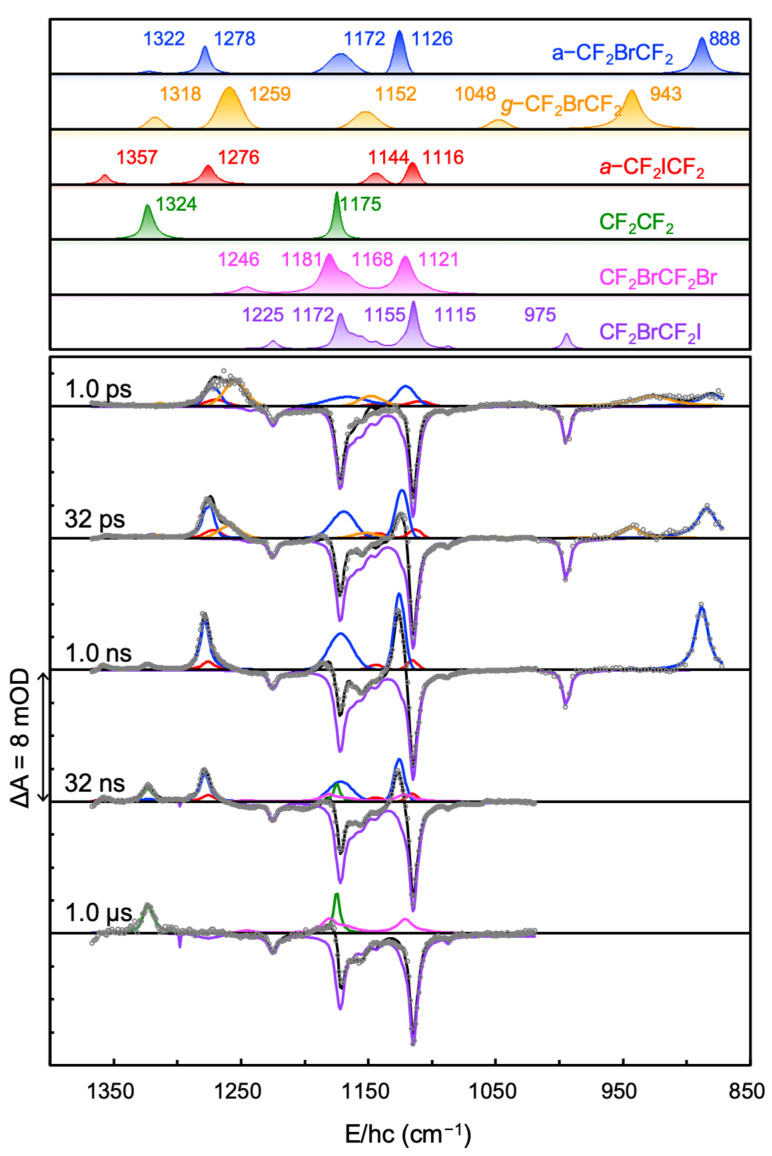
(Upper panel) Basis spectra of CF_2_BrCF_2_I (purple), CF_2_BrCF_2_Br (magenta), CF_2_CF_2_ (green), *a*-CF_2_ICF_2_ (red), *g*-CF_2_BrCF_2_ (orange), and *a*-CF_2_BrCF_2_ (blue) that were used to fit the TRIR spectra of 267-nm-excited CF_2_BrCF_2_I in CCl_4_ at 280 ± 2 K. Positions of the bands are given. (Lower panel) Representative TRIR spectra decomposed into their contributing basis spectra (color-coded) are shown in the upper panel. Data (open circles) were well reproduced by the sum (black lines) of the basis spectra.

When CF_2_BrCF_2_I is excited by UV light, it dissociates I or Br atoms, producing CF_2_BrCF_2_ or CF_2_ICF_2_ radicals, respectively [4,9,10,15]. As mentioned, because 82 ± 3% (18 ± 3%) of the excited CF_2_BrCF_2_I in CCl_4_ at 267 nm is promoted to the antibonding of the C−I (C−Br) bond, the majority of the produced radicals are expected to be CF_2_BrCF_2_. As shown in our previous TRIR spectroscopic experiment on CF_2_BrCF_2_I in the time range of 0.3−320 ps, the TRIR spectra consisted of the spectra of three nascent radicals (*a*-CF_2_BrCF_2_, *g*-CF_2_BrCF_2_, and *a*-CF_2_ICF_2_) and the inverted spectrum of CF_2_BrCF_2_I for the bleach signal [15]. The final photoproducts were 50 ± 3% CF_2_CF_2_ and 50 ± 3% CF_2_BrCF_2_Br, which were produced via secondary reactions of the reaction intermediates. Therefore, the TRIR spectra should include the spectra of the reactant (CF_2_BrCF_2_I), final products (CF_2_CF_2_ and CF_2_BrCF_2_Br), and reaction intermediates (*a*-CF_2_BrCF_2_, *g*-CF_2_BrCF_2_, and *a*-CF_2_ICF_2_). The TRIR spectra were globally fitted using the basis spectra of CF_2_BrCF_2_I, CF_2_CF_2_, CF_2_BrCF_2_Br, *a*-CF_2_BrCF_2_, *g*-CF_2_BrCF_2_, and *a*-CF_2_ICF_2_, as shown in the upper panel of Figure 4. The basis spectra of CF_2_BrCF_2_I, CF_2_CF_2_, and CF_2_BrCF_2_Br were obtained from equilibrium FTIR measurements, and those of *a*-CF_2_ICF_2_, *a*-CF_2_BrCF_2_, and *g*-CF_2_BrCF_2_ were obtained from our previous experiments [8,15]. The decomposition of CF_2_BrCF_2_I and CF_2_BrCF_2_Br into their conformer-specific spectra was not necessary for the fitting because their basis spectra have contributions from both conformers, and these contributions were maintained throughout the experiment. As shown in Figure 2c and the lower panel of Figure 4, the TRIR spectra were well reproduced by the sum of the basis spectra for CF_2_BrCF_2_I, CF_2_CF_2_, CF_2_BrCF_2_Br, *a*-CF_2_BrCF_2_, *g*-CF_2_BrCF_2_, and *a*-CF_2_ICF_2_ shown in the upper panel of Figure 4. The time-dependent amplitude changes in the basis spectra were obtained by global fitting of the TRIR spectra. As mentioned, the magnitude of the bleach spectrum did not change throughout the experimental time span, implying that there was no rebinding of the dissociated halogen atom with its counter radical for up to 10 μs. The amplitudes of the remaining five basis spectra revealed rich kinetic information related to the reaction dynamics of the 267-nm-excited CF_2_BrCF_2_I in CCl_4_ at 280 K.

**Figure 5:**
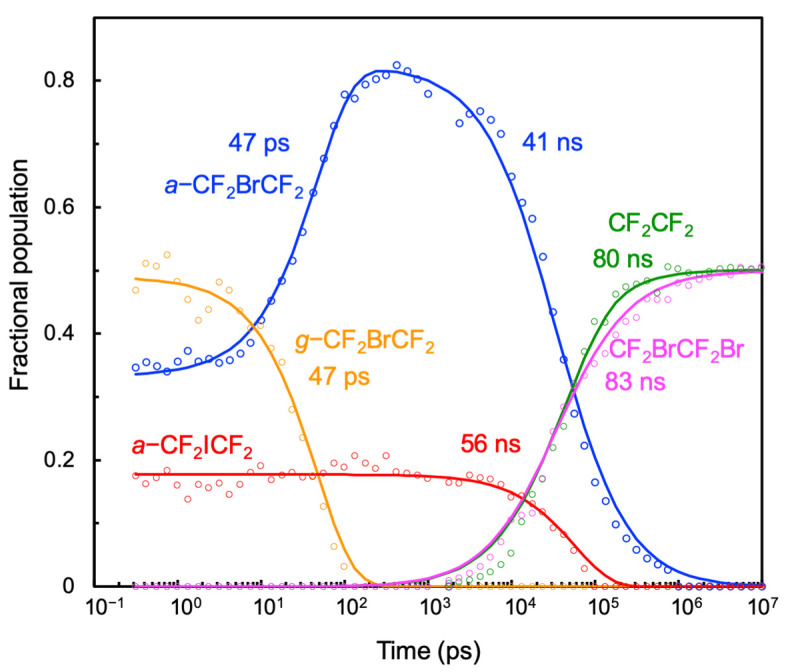
Time-dependent fractional population changes in CF_2_CF_2_ (green), CF_2_BrCF_2_Br (magenta), *a*-CF_2_BrCF_2_ (blue), *g*-CF_2_BrCF_2_ (orange), and *a*-CF_2_ICF_2_ (red), which were derived from the time-dependent amplitude changes of the basis spectra of the corresponding compounds (see text). Time constants for the changes are also shown. Data (open circles) are reproduced by the kinetic scheme (Scheme 1, see text) introduced to describe the time-dependent fractional population changes by adjusting rate constants.

The amplitude of a basis spectrum (*amp*) is related to the population of the corresponding compound by amp ∝ ε×n [15,19], where *ε* and *n* represent the integrated extinction coefficient and population of the compound, respectively. The integrated extinction coefficient (*ε*) of *a*-CF_2_BrCF_2_, *g*-CF_2_BrCF_2_, and *a*-CF_2_ICF_2_ were obtained from our previous measurements [8,15], and those of CF_2_BrCF_2_I, CF_2_CF_2_, and CF_2_BrCF_2_Br were determined from equilibrium FTIR measurements. Time-dependent fractional population changes of CF_2_BrCF_2_I, CF_2_CF_2_, CF_2_BrCF_2_Br, *a*-CF_2_BrCF_2_, *g*-CF_2_BrCF_2_, and *a*-CF_2_ICF_2_ were derived from time-dependent amplitude changes for the basis spectra of the corresponding compounds using the relation, n ∝amp∕ε. As shown in Figure 5, three nascent radicals (*a*-CF_2_BrCF_2_, *g*-CF_2_BrCF_2_, and *a*-CF_2_ICF_2_) appeared immediately, implying that the photodissociation of CF_2_BrCF_2_I occurred within 0.3 ps [15]. The decay of *g*-CF_2_BrCF_2_ correlated with the growth of *a*-CF_2_BrCF_2_ because *g*-CF_2_BrCF_2_ isomerizes into *a*-CF_2_BrCF_2_ at a time constant of 47 ± 5 ps [15]. Moreover, the decays of *a*-CF_2_ICF_2_ and *g*-CF_2_BrCF_2_ correlated with the growth of CF_2_CF_2_ and CF_2_BrCF_2_Br, implying that these products were formed by the secondary reactions of *a*-CF_2_ICF_2_ and *a*-CF_2_BrCF_2_.

**Scheme 1:**
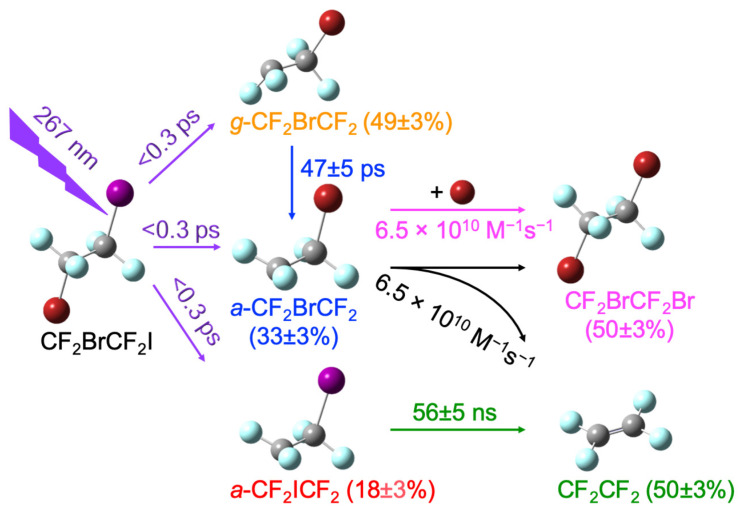
A kinetic scheme used to fit time-dependent fractional populational changes of various compounds appearing when CF_2_BrCF_2_I in CCl_4_ at 280 K is excited at 267 nm. The rate constants and percentage were obtained by globally fitting the TRIR spectra. Gray, light blue, brown, and purple spheres represent carbon, fluorine, bromine, and iodine atoms, respectively. The quoted uncertainties reflect fitting and estimated experimental error.

A kinetic scheme (Scheme 1) was introduced to reproduce the time-dependent fractional population changes, as shown in Figure 5, by optimizing the rate constants of all transitions. As shown in Figure 5, Scheme 1 reproduced the time-dependent fractional population changes.

Scheme 1 indicates that all the excited CF_2_BrCF_2_I at 267 nm dissociated one halogen atom to produce CF_2_ICF_2_ or CF_2_BrCF_2_ (18 ± 3% *a*-CF_2_ICF_2_, 33 ± 3% *a*-CF_2_BrCF_2_, and 49 ± 3% *g*-CF_2_BrCF_2_) [15]. Almost all nascent *g*-CF_2_BrCF_2_ isomerizes into *a*-CF_2_BrCF_2_ with a time constant of 47 ± 5 ps [15]. All of the nascent *a*-CF_2_ICF_2_ undergoes secondary dissociation to produce CF_2_CF_2_ + I with a time constant of 56 ± 5 ns. The nascent *a*-CF_2_ICF_2_ obtained from CF_2_ICF_2_I in CCl_4_ undergoes secondary dissociation with a time constant of 5.5 ns [8]. Although a secondary dissociation time of 56 ns for *a*-CF_2_ICF_2_ is less than that observed previously, it clearly demonstrates that the secondary dissociation of the C−I bond is feasible and can occur on the nanosecond time scale in solution.

The decay of *a*-CF_2_BrCF_2_ was correlated with the growth of the final products, CF_2_CF_2_ and CF_2_BrCF_2_Br. The possible reactions of *a*-CF_2_BrCF_2_ to produce the final products are as follows.

*a*-CF_2_BrCF_2_ + *a*-CF_2_BrCF_2_ → CF_2_CF_2_ + CF_2_BrCF_2_Br (1)

*a*-CF_2_BrCF_2_ + Br → CF_2_BrCF_2_Br (2)

*a*-CF_2_BrCF_2_ → CF_2_CF_2_ + Br (3)

(a) If reaction (1) does not occur, reactions (2) and (3) should proceed, resulting in the formation of the two products, CF_2_BrCF_2_Br and CF_2_CF_2_. Reaction (2), which produces CF_2_BrCF_2_Br, should proceed with a rate constant of (1.6 ± 0.3) × 10^11^ M^−1^s^−1^, and reaction (3), which produces CF_2_CF_2_, with a time constant of 130 ± 5 ns.
(b) As the time constant of 130 ns for the secondary dissociation of *a*-CF_2_BrCF_2_ (reaction (3)) is too high to be realistic (vide infra), reaction (1) should occur to produce CF_2_CF_2_.
(c) Reaction (1) produces equal amounts of CF_2_CF_2_ and CF_2_BrCF_2_Br, and the secondary dissociation of *a*-CF_2_ICF_2_ produces CF_2_CF_2_ (18% of the reactant), resulting in the final products CF_2_BrCF_2_Br (41%) and CF_2_CF_2_ (59%) if reaction (1) is the only one to proceed. Reaction (2) should proceed in addition to reaction (1) to give the observed final products (50 ± 3% CF_2_CF_2_ and 50 ± 3% CF_2_BrCF_2_Br).
(d) Furthermore, if *a*-CF_2_BrCF_2_ reacts bimolecularly with itself (reaction (1)), it can also react with Br atoms in solution (reaction (2)).

Therefore, reactions (1) and (2) were allowed to fit the fractional population changes of *a*-CF_2_BrCF_2_, CF_2_CF_2_, and CF_2_BrCF_2_Br, resulting in rate constants for the BR of *a*-CF_2_BrCF_2_ with itself (reaction (1)) and the Br atoms (reaction (2)), which were approximately the same, (6.5 ± 0.3) × 10^10^ M^−1^s^−1^. The recovered BR rate constant is closer to the calculated diffusion-limited rate constant of 7 × 10^9^ M^−1^s^−1^ than the obtained value of (1.6 ± 0.3) × 10^11^ M^−1^s^−1^, assuming that only reaction (2) produces CF_2_BrCF_2_Br.

The excess energy in the nascent radicals (*a*-CF_2_ICF_2_, *a*-CF_2_BrCF_2_, and *g*-CF_2_BrCF_2_) produced during the photodecomposition of CF_2_BrCF_2_I was thermalized with a time constant of 15 ± 3 ps [15], which is consistent with the thermalization time constant observed in other reactions in solution [8,19]. The solvent acts as an energy sink for the excess energy of a molecule in solution. All nascent radicals were thermalized with a time constant of 15 ± 3 ps after the photodecomposition of CF_2_BrCF_2_I in CCl_4_, indicating that the observed reactions became thermal within tens of picoseconds after the photodecomposition. Therefore, the radicals should gain the energy required for the secondary bond dissociation that occurs within nanoseconds or longer. The bond dissociation energy is supplied by the solvent, indicating that the solvent becomes an energy source as well as an energy sink. As the solvent can act as an energy sink and an energy source, secondary dissociation at a longer duration than thermalization is a characteristic of the reaction in solution. For the secondary dissociation of the C−X bond, the activation energy should be as high as the C−X bond energy. As the C−Br bond energy of 22.3 ± 2.5 kcal/mol is 15.2 kcal/mol higher than the C−I bond energy of 7.1 ± 2.5 kcal/mol [7], the secondary dissociation of the C−Br bond is expected to take approximately 10^12^ times longer than that of the C−I bond, based on the Arrhenius equation for the rate constant at room temperature. Considering that the secondary dissociation time of thermalized *a*-CF_2_ICF_2_ is 56 ns, it is unlikely that the secondary dissociation of Br from thermalized a-CF_2_BrCF_2_ will take 130 ns. 

The Gibbs free energy of *a*-CF_2_BrCF_2_Br was calculated to be 0.91 kcal/mol more stable than that of *g*-CF_2_BrCF_2_Br. Therefore, when thermalized, 72% of CF_2_BrCF_2_Br is in the *anti*-conformer at 280 ± 2 K, while almost all (97%) the CF_2_BrCF_2_ radicals are *a*-CF_2_BrCF_2_. Although most CF_2_BrCF_2_Br is produced by the BR of *a*-CF_2_BrCF_2_, the produced CF_2_BrCF_2_Br is in both *anti* and *gauche* conformers in the equilibrium distribution. Based on the rotational isomerization time of 47 ps for CF_2_BrCF_2_ and the calculated rotational barriers [15], that of CF_2_BrCF_2_Br in CCl_4_ at 280 K was estimated [15] to be 12 ns using the calculated Gibbs free energy for the rotational activation. The Gibbs free energy was found to be 6.21 (7.12) kcal/mol for *gauche*-to-*anti* (*anti*-to-g*auche*) transition by the DFT method using ωB97X-D/aug-cc-pVTZ. As shown in Figure 5, CF_2_BrCF_2_Br was produced with a time constant of 83 ns, which is longer than the estimated rotational isomerization time. Thus, CF_2_BrCF_2_Br was produced in the equilibrium distribution, even though the reactant was mainly the *a*-CF_2_BrCF_2_ radical.

## 3. Materials and Methods

### 3.1. Time-Resolved Mid-IR Spectroscopy

The TRIR spectrometer is based on a commercial Ti:sapphire oscillator/amplifier system (Spitfire Ace, Spectra Physics, Milpitas, CA, USA) that generates 800 nm, 110 fs pulses with a repetition rate of 2 kHz [8,19]. Half of the amplified femtosecond pulses were sent to a third-harmonic generator (THG), and the other half were sent to a homemade linear optical parametric amplifier (OPA). The THG produced 267 nm, 160 fs pulses with 10 μJ of energy. The OPA produced near-IR signal and idler pulses, which were difference frequency mixed in a 1-mm-thick GaSe to generate tunable mid-IR pulses with a duration of 120 fs, 160 cm^−1^ spectral width, and 1 μJ of energy. The sample was excited by a portion of the 267 nm pulse and probed by a small fraction (~10 nJ) of the mid-IR pulse. The 267 nm excitation pulses were optically delayed up to 1 ns relative to the mid-IR probe pulses by a computer-controlled translation stage (M-415. PD, PI, Karlsruhe, Deutschland). The translation stage was impractical for optical delays beyond 1 ns because of the long physical distances for the stage. Thus, the excitation pulses were replaced with the nanosecond pulses (267 nm, 2.5 ns, and 20 μJ) produced by a commercial nanosecond tunable laser (NT240, EKSPLA, Vilnius, Lithuania) based on an optical parametric oscillator. These nanosecond excitation pulses were synchronized with the femtosecond probe pulses using an electronic digital delay generator (DG535, Stanford Research Systems, Sunnyvale, CA, USA), which produced an optical delay beyond 1 ns. The angle between the linearly polarized probe and the excitation pulses was set as the magic angle (54.7°) to obtain an isotropic absorbance proportional to the population of the corresponding compounds free from molecular rotation. The energy of the excitation pulse was reduced to 1–2 μJ to ensure linear absorbance and to minimize thermal lensing, which causes high background absorption on the nanosecond time scale due to solvent heating [21,22,23,24,25]. To measure the spectral region beyond the spectral width (160 cm^−1^) of the probe pulses, the experiment was repeated with probe pulses centered at 1300, 1230, 1160, 1080, or 950 cm^−1^, and the data were combined to obtain a broad spectrum. The probe pulses were passed through the sample and were detected by a 1 × 128-pixel MCT array detector (MCT-8-128, InfraRed Associates, Stuart, FL, USA) for wavenumbers > 1000 cm^−1^ or a 1 × 64-pixel MCT array detector (MCT-16-64, InfraRed Associates, Stuart, FL, USA) for wavenumbers < 1000 cm^−1^ in a 320 mm monochromator (HR320, Horiba, Miami, FL, USA) with a grating of 100, 75, or 50 lines/mm. The 1 × 64-pixel MCT array detector was custom-made to enhance spectral responsivity at longer wavelengths (<1000 cm^−1^). The resulting spectra have a spectral resolution of 0.9–1.2 cm^−1^ for the spectral regions > 1000 cm^−1^ and 2.7 cm^−1^ for spectral regions < 1000 cm^−1^. The instrument response function was determined by the transient absorption of the Si wafer and was 0.2 ps in the subnanosecond experiment (0.3 ps–1 ns) and 2.5 ns in the nanosecond experiment (1 ns–10 μs). The spectra were collected at pump-probe delay times ≥ 0.3 ps to avoid signal complications due to pump-probe overlap [26,27,28]. 

### 3.2. Sample Preparation

CF_2_BrCF_2_I was purchased from Alfa Aesar, and CCl_4_ was obtained from Sigma-Aldrich and used without further purification. A flowing sample cell consisting of two 2-mm-thick BaF_2_ windows and a 100-μm-thick Teflon spacer was connected to a peristaltic tubing pump (Masterflex L/S, Cole-Parmer, Vernon Hills, IL, USA), refreshed with 50 mM CF_2_BrCF_2_I in CCl_4_ fast enough for each probe pulse at a repetition rate of 2 kHz. Concentration and path length were set to obtain the maximum transient absorption signal in the spectral region of interest. A sufficiently large volume of the sample was used to maintain the decrease in the sample concentration as <2% due to the photoreaction of CF_2_BrCF_2_I by 267 nm excitation. The temperature of the sample was maintained at 280 ± 2 K by passing the coolant through the sample mount block. The low temperature was beneficial in minimizing the noise in the TRIR spectra caused by the temperature gradient in the probe spot of the sample induced by the pump energy (i.e., thermal lensing) appearing on the nano- to microsecond time scale [21,22,23,24,25]. To ensure the integrity of the samples, equilibrium UV–Vis and FT-IR spectra were collected before and after the TRIR experiments.

### 3.3. Computational Details

All quantum-chemical calculations were performed using the Gaussian 09 software (Revision D.01). The DFT method with the ωB97X-D (long-range corrected hybrid density functionals with damped atom–atom dispersion corrections) functional was used for molecular geometry optimization and the energies of the optimized compounds. ωB97X-D has been reported to yield satisfactory accuracy in thermochemistry, kinetics, and noncovalent interactions [29,30]. The aug-cc-pVTZ basis set was used for the C, F, and Br atoms, and the aug-cc-pVTZ-PP basis set was used for the I atom. Solvent effects were incorporated into the polarizable continuum model using the integral equation formalism variant. 

## 4. Conclusions

In this study, the photodissociation dynamics of CF_2_BrCF_2_I in a CCl_4_ solution were determined over a broad time span, encompassing the entire reaction, after excitation at 267 nm using TRIR spectroscopy. UV excitation led to ultrafast dissociation of I or Br atoms, resulting in CF_2_BrCF_2_ and CF_2_ICF_2_ radicals, respectively, which further proceeded with conformer-specific reactions. CF_2_BrCF_2_ was the major photofragment with both conformers of *a*-CF_2_BrCF_2_ (33 ± 3%) and *g*-CF_2_BrCF_2_ (49 ± 3%), while *a*-CF_2_ICF_2_ (18 ± 3%) was the minor one. The less stable *g*-CF_2_BrCF_2_ was isomerized with a time constant of 47 ± 5 ps into *a*-CF_2_BrCF_2_. *a*-CF_2_BrCF_2_ further reacted bimolecularly with itself at a diffusion-limited rate to produce CF_2_BrCF_2_Br and CF_2_CF_2_, or with the dissociated Br atom to form CF_2_BrCF_2_Br. However, no secondary dissociation of C−Br in *a*-CF_2_BrCF_2_ was observed. In contrast, *a*-CF_2_ICF_2_ underwent simple secondary dissociation of the I atom with a time constant of 56 ± 5 ns to produce CF_2_CF_2_ + I. The final products contained 50 ± 3% CF_2_BrCF_2_Br and 50 ± 3% CF_2_CF_2_. Structure-sensitive TRIR spectroscopy allowed us to determine the entire reaction dynamics of photoexcited CF_2_BrCF_2_I in a CCl_4_ solution, including the conformer-specific dynamics of the CF_2_BrCF_2_ radicals.

## Data Availability

Not applicable.
